# Different Markers of Semantic–Lexical Impairment Allow One to Obtain Different Information on the Conversion from MCI to AD: A Narrative Review of an Ongoing Research Program

**DOI:** 10.3390/brainsci14111128

**Published:** 2024-11-08

**Authors:** Davide Quaranta, Camillo Marra, Maria Gabriella Vita, Guido Gainotti

**Affiliations:** 1 Neurology Unit, Department of Science of Elderly, Neuroscience, Head and Neck and Orthopaedics, Fondazione Policlinico A. Gemelli, IRCCS, 00168 Rome, Italy; 2 Memory Clinic, Department of Science of Elderly, Neuroscience, Head and Neck and Orthopaedics, Fondazione Policlinico A. Gemelli, IRCCS, 00168 Rome, Italy; 3 Department of Neuroscience, Catholic University of Sacred Heart, Fondazione Policlinico A. Gemelli, Istituto di Ricovero e Cura a Carattere Scientifico, 00168 Rome, Italy

**Keywords:** semantic memory disorders, conversion from MCI to AD, semantic and phonemic fluency tasks, phonemic-semantic fluency discrepancy, word typicality

## Abstract

**Background:** In this narrative review, we have surveyed results obtained from a research program dealing with the role of semantic memory disorders as a predictor of progression from mild cognitive impairment (MCI) to Alzheimer’s disease (AD). **Objectives:** In this research program, we have taken into account many different putative markers, provided of a different complexity in the study of the semantic network. These markers ranged from the number of words produced on a semantic fluency task to the following: (a) the discrepancy between scores obtained on semantic vs. phonemic word fluency tests; (b) the presence, at the single-word level, of features (such as a loss of low typical words on a category verbal fluency task) typical of a degraded semantic system; or (c) the presence of more complex phenomena (such as the semantic distance between consecutively produced word pairs) concerning the organization of the semantic network. In the present review, all these studies have been presented, providing separate subsections for (a) methods, (b) results, and (c) a short discussion. Some tentative general conclusions have been drawn at the end of the review. We found that at baseline all these markers are impaired in MCI patients who will later convert to AD, but also that they do not necessarily show a linear worsening during the progression to AD and allow one to make different predictions about the time of development of AD. Our conclusions were that, rather than searching for the best marker of conversion, we should use a range of different markers allowing us to obtain the information most appropriate to the goal of our investigation.

## 1. Introduction

Alzheimer’s disease (AD) is responsible for the majority of cases of dementia, which affects 5.4% of subjects over 65 and whose prevalence further increases with age [1]. Clinical AD is often preceded by a phase called mild cognitive impairment (MCI) in which there are complaints and objective impairments in one or more cognitive domains, but activities of daily living (ADL) are preserved [2]. Mild cognitive impairment is, however, a rather heterogeneous category because Winblad et al. [3] have shown that when subjects included in this category are followed over time, some of them progress to AD and other types of dementia, but others are stable or can even recover. To clarify this problem, some authors [3,4] suggested a distinction among four types of MCIs: (a) amnestic single domain; (b) amnestic multiple domain; (c) non-amnestic single domain; and (d) non-amnestic multiple domain. Other investigations [5,6,7,8,9,10] tried to determine the neuropsychological aspects of MCI patients that best predict conversion from MCI to AD. These investigations showed that delayed recall is the best method of identifying MCI patients at higher risk of converting to AD. These results are consistent with the pioneering observations of Braak and Braak [11], who showed that the first elementary lesions of AD (and, in particular, the neurofibrillary tangles) involve the entorhinal cortex and hippocampus. These lesions disconnect the Papez circuit from information coming from the outside world through the entorhinal cortex, hindering the formation of new episodic memories. It is, therefore, reasonable to expect that episodic memory disorders may characterize the early stages of AD and the amnesic forms of MCI (aMCI). Clinical observations and neuropsychological research have shown, however, that episodic memory disorders, which concern the memories of specific events or facts, unique to each person, are not the only form of long-term memory disturbances that can be found in patients with Alzheimer’s disease (AD), because a second kind of disorder concerns semantic memory, which encompasses general knowledge about the world, concepts, facts, and information that are not tied to personal experience. The clinical data that had raised the problem of a semantic–lexical impairment in AD patients was the observation that anomia and other naming disorders are evident in AD since the initial phases of the disease [12,13,14]. We became, however, fully aware of the complexity and the implications of these clinical observations only after having made a careful review of the literature concerning the neuropsychological predictors of conversion from mild cognitive impairment (MCI) to AD [15]. While reviewing these clinical and experimental investigations, we realized, in fact, that several disorders of semantic memory (and particularly those found during tasks of categorical fluency) are observed at baseline both in MCI patients converting to AD and in normal elderly subjects who will later develop dementia. The most sizeable of these disorders consists of a dissociation between results obtained on semantic and phonological word fluency tasks. This dissociation was first reported by Monsch et al. [16], who compared the performances of 89 patients with AD and 53 elderly normal control subjects on four verbal fluency tasks (category, letter, first names, and supermarket fluency). These authors showed, in fact, that the greatest degree of discrimination between patients with AD and control subjects was obtained on the category fluency task, whereas the least accurate discrimination was found in the letter fluency tasks. They concluded that this asymmetry was due to the dependence of category fluency from semantic memory, which deteriorates in the early stages of AD. This conclusion was afterwards supported by the observation that this dissociation can also be found in normal elderly subjects who will later develop AD and by investigations evaluating the intrinsic characteristics of words produced by AD patients during a semantic fluency task. As for the first point, Murphy et al. [17] examined the difference between phonemic and semantic fluency tasks in normal controls, amnestic forms of MCI (aMCI), and AD patients and found a greater impairment in semantic, relative to phonemic, fluency tasks, in controls versus aMCI versus AD patients. Other authors observed that words produced by AD patients during a semantic fluency task showed semantic–lexical features typical of a degraded semantic system, such as high typicality [18] and an early age of acquisition [19]. More complex manifestations, such as a strategic search throughout semantic categories, the clustering of words into subcategories, and switching from a subcategory to another one, have also been investigated in AD patients (e.g., [20,21]). The clustering observed in semantic (category) fluency tasks consists of the tendency to produce sequences of words belonging to the same semantic subcategory, whereas switching involves shifting from one subcategory (cluster) to another one. These phenomena, linked to the organization and functioning of the semantic memory at a level higher than that of the single lexical items, can be observed when the semantic fluency tasks require the recall of words from a rather broad category, such as “animals”. In this case, subjects can provide sequences of names of animals belonging to narrower subcategories (e.g., giving first the names of “ferocious wild animals” followed by those of “farm animals”, or the names of “mammals” followed by those of “birds” and so on). Since the tendency to cluster could be linked to the semantic distance between the components of the semantic network, the reduction in AD patients’ clustering and switching abilities had been considered as further proof of the disruption of semantic memory in these patients. However, not all the observations that supported the existence of a semantic memory defect in the prodromal stages of AD were systematically confirmed by other investigations. For instance, even if most studies have shown a poorer semantic than phonemic fluency in MCI patients than in normal controls (e.g., [22,23,24]), other investigations (e.g., [25,26]) have found no discrepancy between the scores obtained in phonemic and semantic verbal fluency tasks by individuals with aMCI and healthy controls.

Furthermore, passing from the factual to the theoretical controversies, it was not clear if the semantic deficits observed in patients with AD (and in aMCI patients who converted to AD) were due to a degradation in semantic representations, [13,14], an impairment in the strategic processes needed to access these representations [27], or to a disruption of semantic control functions [28]. For instance, Troyer et al. [20] suggested that the tendency to cluster could mainly measure the integrity of the semantic store, whereas the tendency to switch could specifically measure the integrity of executive functions; Reverberi et al. [21] showed that the clustering index is not sensitive to semantic impairment and that the switching index is disrupted in patients with executive defects resulting from frontal lobe degeneration.

For all these reasons, we thought that it could be interesting to explore, in more detail, various aspects of the semantic–lexical disorders that are observed in aMCI patients converting to AD, focusing our attention on four different and complementary measures, concerning, respectively, (a) the number of words produced during category fluency tasks; (b) the dissociation between results obtained during semantic/category and phonological fluency tasks; (b) the intrinsic characteristics of words produced during a category fluency task and (c) the semantic distance between word pairs consecutively produced by aMCI and AD patients during a categorical verbal fluency test.

In order to provide a synthetic representation of this complex line of research, we have reported in a table some essential data concerning the study sample, methodology, and results of each investigation included in this narrative review (Table 1).

## 2. Relationship Between the Presence of Markers of Semantic Deterioration at the Single Word Level and Prediction of Conversion from Amnesic Mild Cognitive Impairment to Alzheimer’s Disease

In the first study of our research program [29], we tried to investigate the relationship between the presence in aMCI subjects of markers of semantic deterioration at the single-word level and the prediction of conversion from aMCI to AD during a 2-year follow-up period.

Four groups of subjects were enrolled in this study: the first two groups were composed of 60 patients who fulfilled the current clinical criteria for aMCI [19] and 20 patients who fulfilled the current criteria for probable AD [34] whereas the last two groups were composed of healthy subjects. Twenty-five of them (“young cognitively normal” yCN) were aged between 25 and 50 years and 20 (“old cognitively normal” oCN) were aged between 65 and 75 years. Conversion to dementia was evaluated after a 24-month follow-up. From a neuropsychological point of view, the patients were assessed with the MMSE and with a complex Mental Deterioration Battery [35]. Language was explored with tasks evaluating naming (of objects and actions), phonological verbal fluency [35] and semantic verbal fluency [36]. In the semantic verbal fluency test, subjects were asked to produce in one minute as many names as possible of items belonging to the categories “birds” and “furniture”. A purely lexical (frequency) and a lexical–semantic (typicality) measure of words produced on the semantic fluency task were taken into account in aMCI patients. The frequency of use (FoU) of each word was scored with “Lessico di Frequenza dell’Italiano Parlato” [37]. Typicality (Ty) indices were obtained by an independent sample of 80 normal subjects, aged 25–75, who were asked to indicate on a Likert scale ranging from 1 (least typical) to 7 (most typical) the degree of typicality of each word produced within the corresponding conceptual category, by subjects of the study groups, according to a previously proposed methodology [38]. Individual scores of word typicality were assessed by computing the harmonic mean of scores provided by the controls. For each subject, the values of Ty and FoU were calculated. Since one of the aims of our study was to evaluate the predicting value of the selected lexical–semantic variables with respect to conversion to AD, we divided the groups according to the median value of these variables. Subjects who belonged to the subgroups above or below the median value were then entered in a backward stepwise logistic regression model in which conversion to AD was set as the dependent variable. Variables that displayed a significant difference between converters and non-converters during univariate tests were also entered into this logistic regression model.

A comparison of the results obtained by the four groups of subjects enrolled in this study on the linguistic variables considered showed that the frequency of words produced in the semantic fluency task did not differ across groups, whereas the typicality of words was significantly different. No difference was found between the mean typicality of words produced by aMCI and AD patients, but both groups generated words of a higher typicality than control subjects. This tendency of aMCI and AD patients to produce very few non-typical members of various categories points to an impoverishment of their semantic memory.

Furthermore, the high typicality of the words produced on the semantic fluency test was a good predictor of conversion from aMCI to AD, because, after a 24-month follow-up, 20 subjects (33% of the aMCI sample) converted to dementia, fulfilling clinical criteria for probable AD. Fifteen (50%) of these subjects belonged to the high-typicality subgroup, while the remaining five (16%) belonged to the low-typicality subgroup. This difference was statistically significant (χ^2^ 7.500; *p* = 0.006), confirming that the tendency to produce almost only typical (and very few non-typical) members of various categories points to an impoverishment of semantic memory.

## 3. Standardization and Study of the Clinical Validation of a New Test Assessing Semantic Verbal Fluency

During our study on the relationships between the frequency and typicality of words produced by aMCI patients on categorical/semantic fluency tasks and conversion of these patients to AD, we realized that there are few normative studies on word typicality for categorical tasks and that the existing data may rapidly result in being too old to be reliable (see [39] for a discussion about this issue). We therefore decided to standardize a new categorical/semantic verbal fluency test, which should include the categories “birds” and “articles of furniture” (BAF) [30] on the basis of three main considerations: (a) a selective impairment of birds/living and furniture/non-living categories has been often reported in studies of patients with different anatomical sites of brain damage ([40,41,42] and see [43] for a recent review of studies concerning this issue in AD patients); (b) earlier studies on normal subjects have shown that the mean number of items produced in the same amount of time for “birds” and “articles of furniture” is very similar [25], permitting a simpler confrontation of performances when the living/non-living dichotomy is explored; (c) both these categories are quite narrow, and this makes the exploration of typicality easier, since subjects should produce few very typical exemplars, highly shared among normal subjects, and an higher proportion of less typical exemplars.

The test was standardized on a sample of 268 subjects aged 40 years or more. The raw scores obtained on “birds,” “furniture,” and total BAF scores were put down as dependent variables into linear regression models in which age, education, and gender were entered as predictors. The typicality of lexical entries was evaluated following the methodology of Battig and Montague [44], which was recently reassessed by Van Overschelde et al. [39]. The clinical validation was conducted on 106 subjects affected by aMCI, 178 patients affected by mild AD, and 114 affected by moderate forms of Alzheimer’s disease.

Both age and education influenced the total BAF score; the percentage of subjects with pathological scores on BAF strengthened from aMCI (19%) to mild (45.5%) and moderate (71.1%) AD and the analysis of the receiver operating characteristic curves showed that the BAF scores are highly reliable in distinguishing healthy subjects from aMCI and AD patients.

## 4. The Capacity of the Phonemic-Semantic Verbal Fluency Discrepancy to Detect aMCI Patients Who Will Convert to AD

In the introductory section of this review, we have seen that, even if most studies have shown a poorer semantic than phonemic fluency in MCI subjects than in normal controls (e.g., [22,23,24]), some investigations (e.g., [25,26]) have failed to find similar phonemic–semantic verbal fluency discrepancies between individuals with amnestic MCI and healthy controls. 

Thus, Nutter Upham et al. [25] administering semantic and phonemic verbal fluency tasks to aMCI patients and normal controls had shown that aMCI individuals produced a statistically reduced number of words, relative to cognitively intact older adults, on both phonemic and semantic fluency tasks. These authors obtained correlational data that suggested that all fluency tasks involved defects of executive control more than a disruption of the semantic store. Even more relevant, as for the capacity of the phonemic–semantic verbal fluency discrepancy to detect aMCI patients who will convert to AD, results have been obtained by Rinehardt et al. [26] comparing the performance obtained in the phonemic COWAT test [45] and in the semantic animal fluency test [46] by healthy controls, non-amnestic MCI, aMCI, and AD groups. These authors have, indeed, shown that both MCI groups obtained similar patterns of performance, with a prevalence of the category over the letter fluency in contrast with the prevalence of the letter over the category fluency pattern typically observed in AD. 

### 4.1. The Use of the “Semantic–Phonological Delta (SPD) Index” to Confirm the Capacity of the Phonemic–Semantic Verbal Fluency Discrepancy to Predict the Conversion from aMCI to AD

To clarify the role of the phonemic–semantic verbal fluency discrepancy in anticipating the conversion from aMCI to AD, we planned a new investigation [31], in which we used the BAF as a test of semantic verbal fluency, and we proposed the “semantic–phonological delta (SPD)” index as a reliable measure of the phonemic–semantic verbal fluency discrepancy 

The “semantic–phonological delta (SPD)” index aims to equalize the number of words generated in the phonemic (FAS) and in the semantic (BAF) fluency task by dividing the number of words obtained in each task by the time allowed to produce these words. In the phonemic (FAS) test, participants were, in fact, requested to generate in one minute as many words as possible starting with the letters F, A, and S (three minutes in total), whereas in the semantic (BAF) test they were asked to generate in one minute words belonging to the categories of “birds” and “articles of furniture” (two minutes in total). The “semantic–phonological delta” (SPD) was, therefore, computed as follows:

$$\;SPD=\;\frac{“CFTScore”}{2}-\frac{“FASScore”}{3}$$

In this formula, negative scores correspond to a worse semantic fluency output in comparison to the phonological fluency output.

In our study the SPD was computed on 805 participants, of whom 262 were healthy individuals, 251 aMCI patients, and 292 AD patients. Within these last patients, 178 were mild (MMSE: 18–23) and 114 moderate (MMSE: 10–17). aMCI participants were assessed with a comprehensive neuropsychological battery [35] during a five-year follow-up that was completed by 178 of the initial sample. During the follow-up period, 117 participants progressed to AD, with a median survival time of 42 months.

At baseline, both category (BAF) and phonemic (FAS) fluency scores were significantly worse in mild and moderate AD than in healthy subjects (HS), whereas MCI patients performed similar to HSs on the phonemic task but were significantly worse in the category task. This discrepancy was highlighted by the SPD score.

A survival analysis of aMCI patients who completed the five-year follow-up was made with a univariate Cox proportional hazard model, in which each neuropsychological test was individually set as a predictor of conversion to AD. On this analysis, the SPD had a strong capacity to predict progression to dementia (HR = 0.92; 95%CI = 0.881–0.968; *p* = 0.001), and the same effect was observed for the CFT (HR = 0.91; 95%CI = 0.859–0.956; *p* < 0.001). The highest value of the SPD was obtained in aMCI patients who converted to AD (−2.15), followed by stable aMCI (−1.20), mild AD (−1.15), moderate AD (−0.95), and HS (−0.80).

Taken together, these results show that the phonemic–semantic verbal fluency discrepancy, evaluated with the PSD, is a marker of AD development that reaches its highest value in the first stages of the progression from aMCI to AD. These partly unexpected results cannot be attributed to the low number of patients who entered the study as had been the case for authors [25,26], who did not find a discrepancy between phonemic and semantic verbal fluency scores in patients with amnestic MCI and in normal controls) because the number of healthy and pathological subjects included in our study was very high. 

### 4.2. A Study of the Neuropsychological Predictors of Conversion from aMCI to AD at Different Timepoints

The results obtained in our previous study suggested that it could be interesting to evaluate at the baseline the role that different possible neuropsychological markers could play in predicting progression from MCI to overt dementia. With this aim in mind, we administered in a new study [32] an extensive battery of neuropsychological tests [35] to a large number of consecutively enrolled individuals who met the definition of aMCI [47] and who were followed up every 6 months for 6 years. The sample included 253 MCI patients, with a mean age of 72.95 years, a mean literacy of 10.76 years, and a mean MMSE score of 25.69. During the follow-up, MCI participants underwent, every 6 months, a complete neurological, medical, and neuropsychological assessment. The progression to dementia was assessed at each follow-up visit by a neurologist who did not know the results of the baseline neuropsychological examination. Subjects were diagnosed as AD if they met the clinical criteria for this disease [34] and obtained a CDR score of 1. Subjects who progressed to dementia were divided into three groups based on the time of conversion to AD: fast (during the first 2 years after the baseline), intermediate (after 2–4 years of follow-up), and slow (more than 4 years after the baseline). 

Significant differences were obtained between stable MCI subjects and converters to AD at different timepoints. Stable MCI patients performed better than fast decliners on MMSE, several long-term memory scores, and category verbal fluency tests (CFTs); they performed better than intermediate converters only on MMSE and CFTs and scored better than slow converters only on MMSE and phonological–semantic discrepancy score.

Measures of episodic and semantic memory impairment allowed, therefore, different predictions of the progression to AD according to the time of observation after symptom onset.

Episodic memory scores were significantly lower in fast converters than in stable MCI subjects, whereas the disruption of semantic memory was the only marker that significantly differentiated stable MCI subjects from intermediate and slow converters. In particular, the “semantic–phonological delta” was the only marker, apart from MMSE, that differentiated stable MCI subjects from slow converters.

Both the data and the observation that the predictive value of SPD as a marker of AD development is much higher in the first stages of the conversion to AD than in more advanced stages of the disease progression are, therefore, consistent with those of earlier studies that had taken this problem into account. In particular, they confirm results obtained by authors who had stressed the predictive value of poor vocabulary in participants of the “Nun study” [48] or the decreased number of words used by famous writers in periods in which they were developing Alzheimer’s disease [49], because all of these studies had found reduced semantic achievements many years before the clinical development of AD.

## 5. Can Measures of the Semantic Relations Between Consecutively Produced Word Pairs Help to Evaluate the Degradation of the Semantic System and to Predict Progression to AD in Individuals with Amnesic MCI?

In the introductory section of this review, we have noticed that various lines of research have been followed to clarify the meaning of the semantic memory disorders observed in AD patients and in aMCI patients who were developing Alzheimer’s disease. Besides the low number of words produced in semantic fluency tasks, the discrepancy between the number of words generated in phonemic and semantic fluency tasks and the analysis of the intrinsic (e.g., typicality) characteristics of the words produced in category fluency tasks, a fourth line of research studied more complex phenomena observed in category fluency tasks, such as the tendency to cluster words belonging to the target category into narrower subcategories and to shift from one subcategory to another. Two different accounts, based, respectively, on the structure of the semantic network corresponding to the target category and on the intentional strategies followed during the search within that category, have been given this tendency [20,50]. The first account assumed that items were clustered into subcategories because the distance between the items belonging to these subcategories was shorter than those existing between the items belonging to different categories. Thus, the spread of activation should prioritize items belonging to the same subcategory in comparison to items belonging to different subcategories. Reverberi et al. [21,51] have, indeed, suggested that the semantic proximity between successive word pairs generated in a category fluency task could measure this semantic distance and be an index of the integrity of the semantic network. The second account assumed, on the contrary, that the tendency to cluster was not linked to the structure of the explored semantic network, but to the intentional strategy followed by the individual during the search activity. Investigations that have studied clustering and switching in AD (e.g., [52,53] have documented that in this condition there is a reduced generation of clusters and switches compared to healthy individuals. However, the literature on clustering and switching in semantic verbal fluency often relies upon predetermined clusters (e.g., “farmyard birds”) that may not capture differences between healthy individuals and patients at the early stages of AD. The interest in these findings has been increased by the recent development of several lexical databases, which have collected and systematized lexical entries on the basis of semantic hierarchies. An important tool of this kind is, for instance, the WordNet database [54], which is organized in a hierarchical manner and in which lexical and conceptual links associate words and groups of words. The basic element of WordNet is the synset (set of synonyms), defined by a gloss, namely by a verbal description of the concept to which it refers. Several conceptual relationships are taken into account in this database, making it suitable for the assessment of semantic relationships between different lexical entries. 

Since the semantic distance could be a good index of the semantic network [21,51] we used WordNet in our investigation [33] to check two problems: (a) if the semantic relations between lexical entries generated on a categorical fluency (the BAF) test were different between healthy persons and patients with aMCI, and (b) if this difference was more pronounced in subject who converted to dementia during a 3-year follow-up period.

Two criteria measuring semantic relationships between consecutively produced word pairs (namely path length and extended gloss overlap) were obtained from the Wordnet database. Path length evaluates the semantic relations between consecutively produced word pairs, whereas extended gloss overlap [55] determines the amount of overlap between the glosses defining two different concepts. The number of clusters produced by each subject while performing the BAF test was assessed in 29 healthy persons (HPs) and 34 individuals with aMCI (10 of whom converted to dementia). 

Semantic relations (path length and extended gloss overlap) between consecutively produced word pairs were measured at the baseline on these subjects. The individuals with aMCI who converted to dementia during the follow-up period showed a reduced mean strength of semantic relationships for both categories when the extended gloss overlap measure of semantic relatedness was considered and, only for “pieces of furniture”, when the path length similarity measure was taken into account. These results support the hypothesis of very early impairment of semantic knowledge in Alzheimer’s disease [56] because semantic memory is a component of declarative memory, organized on the basis of context shared by associated lexical items [57], and involves the knowledge of concepts and words.

## 6. Concluding Remarks and Possible Developments

Taken together, the results summarized in this review consistently show that several markers of semantic memory disorders are impaired at baseline in MCI patients who will later convert to AD. These markers are as follows: (1) the number of words generated in a semantic/category word fluency task; (2) a discrepancy between the number of words generated in tasks of semantic vs. phonemic fluency; (3) intrinsic (e.g., typicality) semantic characteristics of single words produced in these tasks; and (4) measures of semantic proximity between successive word pairs, suggesting an impairment of the semantic network. Some of these markers did not necessarily show further worsening from the baseline to the time of conversion to AD. For instance, the discrepancy between the number of words produced in tasks of semantic/categorical vs. phonemic fluency reached its highest value in the first stages of the progression from aMCI to AD, decreasing in the following stages. Other markers, however, such as the tendency to obtain a pathological score on the (BAF) category fluency test showed a progressive worsening during the progression to AD, because this marker grew from aMCI (19%) to mild (45.5%) and moderate (71.1%) AD. One reason for the greater difference in scores in the early stages of the disease could be the fact that lexical word-finding mechanisms were not affected on letter fluency tasks in the initial degeneration stages but gradually deteriorated as the disease progressed, thus decreasing the value of the SPD.

Other more general cognitive disorders linked to the progression of the disease could explain the dissociation between the progressive worsening of the results obtained measuring only the number of words generated in the category fluency task and the lack of advancement documented on the SPD. One of them could be the development of executive disorders, which could impact more on the phonemic than on the semantic fluency tasks [24,58,59] and could, therefore mask the initial semantic–phonemic discrepancies. In any case, it is conceivable that different markers of lexical-semantic impairment may provide various pieces of information depending upon the time at which the assessment is made.

From the theoretical point of view, the results of investigations conducted in the last decade are not conclusive with respect to the alternative between representational and retrieval deficits in MCI and AD (e.g., [13,14]). Even if most of our results can be interpreted as the effect of an early disturbance in semantic representation, the effect of retrieval deficits cannot be excluded. In this sense, the exploration of semantic impairment can be conducted using implicit techniques, such as semantic priming. We have previously reported impaired semantic priming in subjects with MCI who eventually progressed to dementia [60], but further studies are needed, since even the presence and nature of a priming effect in AD is still a matter of debate [61].

Future studies are also warranted with respect to two other aspects of semantic memory in AD and MCI. The first one is represented by the neuroanatomical correlates of these disturbances. The role of medial-temporal regions in semantic memory has been documented [56], together with the evidence that the Default Mode Network may be involved in semantic memory disturbances. The exploration of the relations between the variables described in this review and changes in specific brain regions in AD may provide a contribution to trying to define the neuroanatomical correlates of semantic processing. 

The second aspect is the opportunity to use the features of word production in categorical fluency tasks to explore aspects of semantic memory that could be less affected by concomitant abilities As suggested in previous works on cognitively healthy subjects, lexical variables may indeed be useful indices of the different steps leading to word production [62].

One last consideration could regard future perspectives concerning new applications of the automatized recording of spontaneous speech and fluency tasks, which could permit one to obtain an automatized scoring, and comparative analyses of new markers in a digitalized way. These new applications can pave the way to automatic screening policy on large population studies that will permit a timely case finding of AD in the very early stages of the disease.

## Figures and Tables

**Table 1:** A summary of the reviewed papers.

Study Reference	Study Sample	Methodology	Results
Vita et al., 2014 [29]	60 aMCI 20 AD 25 yCN 20 oCN	Measurement of FoU and typicality in semantic verbal fluency 2-year follow-up	FoU: AD = aMCI = oCN = yCN Typicality AD = aMCI > oCN = yCN Typicality predicted conversion after the 2-year follow-up
Quaranta et al., 2016 [30]	268 CN 106 aMCI 178 mild AD 114 moderate AD	Standardization and normative values of the Birds and Articles of Furniture (BAF) test Normative values for typicality Clinical validation of the BAF	Pathological performances on BAF: 19% in aMCI 45.5% in mild AD 71.1% in moderate ROC curves showed good reliability in distinguishing CN from aMCI and AD patients.
Marra et al., 2021 [31]	262 HS 251 aMCI 292 AD	Semantic–phonological delta (SPD) 5-year follow-up for aMCI	SPD score: converted aMCI: −2.15 stable aMCI: −1.20 mild AD: −1.15 moderate AD: −0.95 HS: −0.80 High predictive value of SPD for conversion to AD
Quaranta et al., 2023 [32]	253 aMCI	Progression classified as fast (<2 years) intermediate (2–4 years) slow (>4 years) 6-year follow-up	Episodic memory significantly lower in fast converters SPD only marker differentiating stable MCI from slow converters
Quaranta et al., 2019 [33]	29 HS 34 aMCI	Assessment of semantic relationship in semantic verbal fluency (path length and extended gloss overlap)	Reduced strength of semantic relationship between consecutive words in aMCI

aMCI: amnesic MCI; yCN: young cognitively normal; oCN: old cognitively normal; HSs: healthy subjects; FoU: frequency of use.

## Data Availability

No new data were created or analyzed in this study. Data sharing is not applicable to this article.
